# Insulin suppresses ghrelin-induced calcium signaling in neuropeptide Y neurons of the hypothalamic arcuate nucleus

**DOI:** 10.18632/aging.100400

**Published:** 2011-11-08

**Authors:** Yuko Maejima, Daisuke Kohno, Yusaku Iwasaki, Toshihiko Yada

**Affiliations:** ^1^ Department of Physiology, Division of Integrative Physiology, Jichi Medical University School of Medicine, 3311-1 Shimotsuke, Tochigi 329-0498, Japan; ^2^ Department of Developmental Physiology, Division of Adaptation Development, National Institute for Physiological Sciences, Okazaki, Aichi 444-8585, Japan

**Keywords:** ghrelin, insulin, neuropeptide Y, arcuate nucleus, insulin receptor, cytosolic Ca2+, hypothalamus

## Abstract

Neuropeptide Y (NPY) neurons in the hypothalamic arcuate nucleus (ARC) play an important role in feeding regulation. Plasma levels of ghrelin and insulin show reciprocal dynamics before and after meals. We hypothesized that ghrelin and insulin also exert reciprocal effects on ARC NPY neurons. Cytosolic Ca^2+^ concentration ([Ca^2+^]_i_) was measured by fura-2 microfluorometry in single neurons isolated from ARC of adult rats, followed by immunocytochemical identification of NPY neurons. Ghrelin at 10^−10^ M increased [Ca^2+^]_i_ in isolated ARC neurons, and co-administration of insulin concentration-dependently suppressed the ghrelin-induced [Ca^2+^]_i_ increases. Insulin at 10^−16^ M, 10^−14^ M, 10^−12^ M and 10^−10^ M counteracted ghrelin action in 26%, 41%, 61% and 53% of ghrelin-responsive neurons, respectively, showing a maximal effect at 10^−12^ M, the estimated postprandial concentration of insulin in the brain. The majority (>70%) of the ghrelin-activated insulin-inhibited neurons were shown to contain NPY. Double-immunohistochemistry revealed that 85% of NPY neurons in ARC express insulin receptors. These data demonstrate that insulin directly interacts with ARC NPY neurons and counteracts ghrelin action. Our results suggest that postprandial increase in plasma insulin/ghrelin ratio and insulin inhibition of ghrelin action on ARC NPY neurons cooperate to effectively inhibit the neuron activity and terminate feeding.

## INTRODUCTION

Ghrelin and insulin are, respectively, orexigenic and anorexigenic hormones regulating feeding behavior [1-3]. Furthermore, insulin and ghrelin regulate energy and glucose metabolism [4, 5]. Insulin and ghrelin are also implicated in learning/memory [6, 7], and insulin action is characteristically related to the Alzheimer disease [7]. All these functions are known to influence aging. In fact, insulin signaling regulates longevity, and ghrelin could promote longevity and serve as an anti-aging hormone [4, 8, 9, 10, 11]. These observations suggest that insulin, ghrelin and their interaction play an important role in regulation of feeding, metabolism and aging.

Prior to meal intake, plasma ghrelin level is elevated and plasma insulin level is low, while following meal intake, plasma ghrelin level decreases and plasma insulin level increases [12]. Accordingly, dramatic reciprocal changes of these hormone levels are produced, possibly contributing to initiation and termination of feeding behavior. Thus, ghrelin and insulin are reciprocally regulated at the level of release. In addition, we hypothesized that ghrelin and insulin could act reciprocally on the target neuron in the feeding center, to further promote the interplay of the two hormones in regulating feeding.

A large majority of neuropeptide Y (NPY) neurons in the hypothalamic arcuate nucleus (ARC) express the ghrelin receptor, growth hormone-secretagogue receptor (GHS-R) [13], and ghrelin directly interacts with and increases cytosolic Ca^2+^ concentration ([Ca^2+^]_i_) in the NPY neurons isolated from ARC [14]. It was shown that ghrelin also increases food intake via vagal afferent-mediated neural transmission to NPY neurons in ARC [15]. Thus, the principal mediator of orexigenic action of ghrelin is the ARC NPY neuron. On the other hand, anorexigenic effect of insulin could also involve the hyothalamic NPY. Inhibition of insulin receptor expression in the hypothalamus causes hyperphagia and increases fat mass [16]. Intracerebroventricular (ICV) injection of insulin inhibits the fasting-induced expression of NPY mRNA in the hypothalamus [17]. Insulin deficient type 1 diabetes exhibits increased expression of NPY and hyperphagia [18]. These reports suggest that the anorexigenic effect of insulin is mediated by inhibition of NPY neurons in the hypothalamus. However, direct effect of insulin on the ARC NPY neurons has not been evidenced. In the present study, we aimed to determine whether insulin directly interacts with ARC NPY neurons and counteracts the stimulatory action of ghrelin. We isolated single neurons from the ARC of rats aged 6 weeks when the feeding function largely matures, measured [Ca^2+^]_i_ to monitor the neuronal activity, and observed direct effects of ghrelin and insulin on [Ca^2+^]_i_. Localization of the insulin receptor in NPY neurons of ARC was studied immunohistochemically.

## RESULTS

### Insulin suppresses ghrelin-induced [Ca^2+^]_i_ increases in isolated ARC neurons

**Figure 1:**
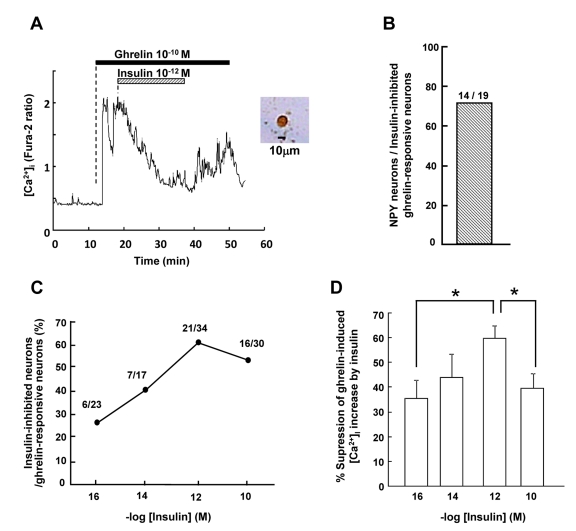
Insulin suppresses ghrelin-induced [Ca^2+^]_i_ increases in ARC NPY neurons (**A**) Administration of 10^−10^ M ghrelin increased [Ca^2+^]_i_ and administration of 10^−12^ M insulin suppressed the ghrelin-induced [Ca^2+^]_i_ increases in a single neuron isolated from ARC. This neuron was subsequently identified as an NPY containing neuron by immunocytochemistry. (**B**) Fourteen of 19 ghrelin-activated insulin-inhibited neurons (74%) were NPY neurons. (**C**) The percentage of insulin-inhibited neurons among ghrelin-activated neurons. (**D**) The amplitude of suppression of ghrelin-induced [Ca^2+^]_i_ increases by insulin is expressed as percent suppression. n=23, 17, 34 and 27 for 10^−16^ M, 10^−14^ M, 10^−12^ M and 10^−10^ M insulin, respectively. Bars in (**D**) represent means ± SE. *p < 0.05.

To determine the direct effect of insulin on NPY neurons, we used a method of the [Ca^2+^]_i_ imaging in isolated single neurons followed by immunocytochemical identification of NPY neurons [14, 19]. Administration of 10^−10^ M ghrelin increased [Ca^2+^]_i_ in the neurons isolated from ARC, confirming previous reports [14, 19]. The ghrelin-induced [Ca^2+^]_i_ increase was markedly suppressed by administration of 10^−12^ M insulin (Figure 1A, left panel) in a neuron that was subsequently shown to contain NPY (Figure 1A, right panel). The majority (14 of 19, 74%) of the ghrelin-activated insulin-inhibited neurons were NPY neurons (Figure 1B). Insulin exerted this effect in a concentration-dependent manner: insulin at 10^−16^ M, 10^−14^ M, 10^−12^ M, and 10^−10^ M suppressed ghrelin-induced [Ca^2+^]_i_ increase in 6 of 23 (26%), 7 of 17 (41%), 21 of 34 (62%), and 16 of 30 (53%) ghrelin-responsive neurons, respectively (Figure 1C). Percent reduction of amplitude of ghrelin-induced [Ca^2+^]_i_ increase by insulin was 35%, 44%, 60% and 40% with insulin at 10^−16^ M, 10^−14^ M, 10^−12^,M and 10^−10^ M, respectively (Figure 1D). Thus, insulin at 10^−12^ M exerted a maximal effect.

### Localization of insulin receptor in NPY neurons of ARC

**Figure 2:**
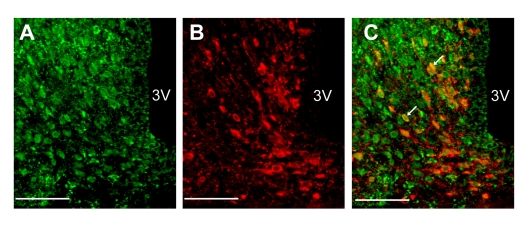
Localization of insulin receptors in NPY neurons in ARC (**A-C**) Fluorescence images for insulin receptor (**A**) and NPY (**B**) and merged image (**C**) in ARC. White arrows in panel C indicate NPY neurons that express insulin receptor. Scale bars in all figures indicate 100 μm.

Double-fluorescence immunohistochemistry showed that the neurons expressing NPY and those expressing insulin receptor were abundantly distributed in the ARC of rat hypothalamic section (Figure 2, A-B). A large majority (85%) of NPY neurons expressed insulin receptors (Figure 2C).

## DISCUSSION

Insulin plays an important role in the central regulation of feeding and metabolism [3, 16], including its action via the hypothalamus to suppress hepatic glucose production [20]. Insulin enters the brain through the blood-brain barrier (BBB) [21]. However, the target neurons of insulin in the hypothalamus have not been fully clarified. ARC is considered the first order center that senses peripheral signals including hormones. Insulin deficient type 1 diabetes exhibits hyperphagia and is associated with increased expression of NPY in ARC [18]. Furthermore, type 2 diabetic Goto-Kakizaki (GK) rats with impaired insulin secretion and action also show hyperphagia and increased expression of NPY in ARC [22]. Hence, the ARC NPY neuron has been suggested as a candidate target for anorexigenic action of insulin. The present study demonstrated that insulin directly interacts with ARC NPY neurons and counteracts the ghrelin action to increase [Ca^2+^]_i_. Since the ghrelin-induced [Ca^2+^]_i_ increase in ARC NPY neurons is coupled to stimulation of food intake [19], the insulin action to counteract ghrelin in ARC NPY neurons is suggested to be linked to inhibition of feeding. Regarding the insulin antagonism against ghrelin, insulin reportedly decreases preproghrelin mRNA expression in proGhrelin-expressing mHypoE-38 neuronal cell line [23].

It has been reported that the plasma insulin concentration increases to a level around 5 × 10^−10^ M after meals [12], and that the peripheral insulin enters the brain through BBB at a rate of 0.046 % [21]. From these reports, the insulin concentration in the brain after meals is estimated to be in a range of 10^−13^ M ~10^−12^ M. In the present study, 10^−12^ M insulin exerted a maximal effect on NPY neurons. Therefore, it is suggested that the inhibition of NPY neurons by insulin takes place most effectively following ingestion of food.

Ghrelin is the only one orexigenic hormone of the peripheral origin, whose plasma level is high before meals and falls after meals. In contrast, plasma level of anorexigenic insulin is low before meals and rises after meals. Thus, the plasma levels of these hormones change in a reciprocal manner. As an underlying mechanism, ghrelin inhibits insulin release from pancreatic islets [5, 24]. In addition to the reciprocal pattern of the plasma levels, the present study has demonstrated that these hormones reciprocally regulate their common target, ARC NPY neurons. It is suggested that at postprandial states, decreased ghrelin level, increased insulin level, and insulin action to counteract ghrelin-induced activation of NPY neurons work in concert to effectively inhibit the NPY neuron activity and terminate feeding.

We previously reported that leptin also suppresses 10^−10^ M ghrelin-induced [Ca^2+^]_i_ increases in ARC NPY neurons in a concentration-dependent manner showing a maximal effect with 10^−12^ M leptin [19]. The estimated physiological concentration of leptin in the brain is around 10^−12^ M [19, 21]. Our present and previous data suggest that postprandial concentrations of insulin and leptin in the brain exert most efficient suppression of ARC NPY neurons, contributing to production of satiety.

In summary, this study demonstrates that insulin directly interacts with ARC NPY neurons and attenuates ghrelin-induced activation of ARC NPY neurons. It is suggested that postprandial increase in plasma insulin/ghrelin ratio and insulin inhibition of ghrelin action in ARC NPY neurons cooperate to effectively inhibit the neuron activity and terminate feeding behavior.

## METHODS

### Animals

Male Sprague-Dawley rats aged 6 weeks were used in the experiments. Animals were maintained on a 12-h light/dark cycle and given conventional food and water ad libitum. Experimental procedures and care of animals were carried out according to the Jichi Medical School Institute of Animal Care and Use Committee.

### Preparation of single neurons from ARC

Single neurons were prepared from the ARC following previous reports [14, 25]. Briefly, the whole ARC was dissected out and incubated with 20 units/ml papain (Sigma Chemical, St. Louis, MO), 1 mM cystein, 0.015 mg/ml deoxyribonuclease, and 0.75 mg/ml BSA for 15 min at 36 °C. The single neurons obtained were distributed onto coverslips and incubated in the humidified chamber at 30 °C for 30 min to 6 h until use.

### Measurement of [Ca^2+^]_i_ and criteria for [Ca^2+^]_i_ responses in single ARC neurons

[Ca^2+^]_i_ was measured by radiometric fura-2 fluorescence imaging as previously reported [14, 26]. Briefly, single neurons on coverslips were incubated with 2 μmol/l fura-2/AM (Dojin chemical, Kumamoto, Japan) for 1 h at room temperature, mounted in chamber and superfused with HKRB at 1 ml/min at 33 °C. Fluorescence images due to excitation at 340 and 380 nm were captured and the ratio (F340/F380) images produced by an Argus-50 system (Hamamatsu Photonics, Hamamatsu, Japan).

When changes in ratio (F340/F380) took place within 5 min after administration of reagents and their amplitudes were more than 0.5 ratio unit, they were considered the responses. Regarding the suppression of ghrelin response by insulin, when insulin decreased the ghrelin-induced [Ca^2+^]_i_ increase by 40% or greater, it was considered the suppression.

### Immunocytochemistry for NPY in single neurons

Immunocytochemistry of NPY neurons were carried out as previously reported [14, 19]. Rabbit anti-NPY antibody (Diasorin, Srillwater, MN, 1:10000) was used for NPY staining. Correlation of [Ca^2+^]_i_ and immunocytochemical data were performed by comparing the images of the phase-contact photographs of the cells subject to [Ca^2+^]_i_ measurements and the immunocytochemical results.

### Dual immunocytochemistry for NPY and insulin receptor in ARC

Colchicine (0.2 mg/15 μl) was ICV injected 1 day before the perfusion. Rat brains were perfused with 4 % parformaldehyde and embedded in paraffin wax. Five μm coronal sections of brain between −1.78 and −2.45 mm from bregma based on the structure of the Brain Maps [27] were cut using a microtome. To retrieve antigenicity, sections were heated by autoclave in 10 mM citrate buffer for 20 min [28]. Sections were immunostained according to standard procedures [26, 28]. Mouse monoclonal antibody against insulin receptor β-subunit (CHEMICON, CA, 1:100) and rabbit anti-NPY antibody (Diasorin, Srillwater, MN, 1:10000) were used as primary antibodies, and Alexa 488 labeled anti-mouse IgG and Alexa 594 labeled anti-rabbit IgG (Molecular probes CA, 1:500) were used as secondary antibodies, respectively. Fluorescence images were acquired with Olympus BX50 fluorescence microscope.

### Solution and chemicals

Measurements were carried out in HKRB solution composed of 129 mmol/l NaCl, 5.0 mmol/l NaHCO_3_, 4.7 mmol/l KCl, 1.2 mmol/KH_2_PO4, 1.8 mmol/l CaCl_2_, 1.2 mmol/l MgSO_4_, and 10 mmol/l HEPES at pH 7.4. Ghrelin (rat) and insulin (porcine) were purchased from Peptides Institute (Osaka, Japan) and Sigma (Tokyo, Japan), respectively.

### Statistical analysis

One-way ANOVA followed by Bonferroni's Multiple Range test were used.

## Abbreviations

ARC: arcuate nucleus
CNS: central nervous system
IR: insulin receptor
NPY: neuropeptide Y
HKRB: HEPES-buffered Krebs-Ringer bicarbonate buffer
ICV: intracerebroventricular
GHS-R: growth hormone secretagogue receptor
BBB: blood brain barrier
